# Loganin, an Iridoid Glycoside, Alleviates Paclitaxel‐Induced Skeletal Muscle Toxicity by Enhancing Mitochondrial Function, Boosting Antioxidant Defenses, and Reducing Cellular Senescence

**DOI:** 10.1002/kjm2.70117

**Published:** 2025-09-27

**Authors:** Yu‐Fan Chuang, Cai‐Rong Wu, Wan‐Hsuan Chang, Ying‐Jung Su, Hung‐Te Hsu, Fan‐Li Lin, Yi‐Ching Lo

**Affiliations:** ^1^ Department of Pharmacology, School of Medicine, College of Medicine Kaohsiung Medical University Kaohsiung Taiwan; ^2^ Graduate Institute of Medicine, College of Medicine Kaohsiung Medical University Kaohsiung Taiwan; ^3^ Department of Anesthesiology Kaohsiung Medical University Hospital Kaohsiung Taiwan; ^4^ Department of Anesthesiology, School of Medicine, College of Medicine Kaohsiung Medical University Kaohsiung Taiwan; ^5^ School of Post‐Baccalaureate Medicine, College of Medicine Kaohsiung Medical University Kaohsiung Taiwan; ^6^ Department of Medical Research Kaohsiung Medical University Hospital Kaohsiung Taiwan; ^7^ Department of Traditional Medicine, School of Medicine, College of Medicine Kaohsiung Medical University Kaohsiung Taiwan; ^8^ Precision Sports Medicine and Health Promotion Center Kaohsiung Medical University Kaohsiung Taiwan

**Keywords:** cellular senescence, loganin, mitochondrial dysfunction, paclitaxel‐induced myotoxicity, skeletal muscle atrophy

## Abstract

Mitochondrial dysfunction and energy imbalance caused by chemotherapy are key contributors to skeletal muscle atrophy, which severely impacts the quality of life in cancer patients. Paclitaxel, a commonly used chemotherapeutic agent, is known to promote muscle wasting and cellular senescence, largely by impairing mitochondrial function. In this study, we investigated the protective role of loganin, a naturally occurring iridoid glycoside, in preventing paclitaxel‐induced damage to skeletal muscle cells. Using C2C12 cells, we assessed whether loganin could counteract the harmful effects of paclitaxel. Our results demonstrated that loganin significantly improved cell viability and protected mitochondrial function, as reflected by better preservation of mitochondrial DNA content, membrane potential, and ATP production, while further enhancing mitochondrial biogenesis through upregulation of PGC‐1α, TFAM, and NRF1. In parallel, loganin activated metabolic regulators SIRT1 and AMPK, while restoring PDK4 expression, suggesting improved energy regulation. Additionally, glycogen levels and myotube morphology were maintained, alongside sustained myosin heavy chain expression. Loganin effectively reduced both cellular and mitochondrial reactive oxygen species and increased antioxidant defenses, including superoxide dismutase activity and glutathione levels. Notably, it also suppressed paclitaxel‐induced senescence and inflammation, as shown by decreased p21 expression, reduced NFκB phosphorylation, and lower levels of *Cdkn1a* and *Il6* as well as reduced SA‐β‐gal staining. Overall, our findings demonstrate that loganin offers comprehensive protection against paclitaxel‐induced skeletal muscle injury by preserving mitochondrial function, supporting metabolic homeostasis, reducing oxidative stress, and limiting senescence. These results highlight the potential of loganin as a preventive adjunctive agent to mitigate chemotherapy‐related muscle toxicity.

## 1 Introduction

Chemotherapy remains an indispensable cancer treatment modality, offering great improvements in survival across many malignancies. Of such drugs, paclitaxel is used extensively due to its robust ability to inhibit proliferation of tumor cells via microtubule stabilization and mitosis arrest [1]. However, clinical utility is generally hampered by off‐target toxicities; notably, in skeletal muscle, paclitaxel induces debilitating side effects such as muscle weakness, fatigue, and atrophy [2, 3]. The symptoms presented are characteristic of chemotherapy‐induced sarcopenia, which seriously impairs physical function, lowers treatment adherence, and ultimately worsens patients' prognosis [4, 5, 6].

One of the main and toxic effects of paclitaxel on skeletal muscle is the drastic impairment of mitochondrial homeostasis. Mitochondria play a central role in the energy metabolism and contractility of muscle. Paclitaxel inhibits their function through decreasing membrane potential, ATP synthesis, and mitochondrial DNA (mtDNA) levels [7, 8, 9], which indicate extreme bioenergetic failure compromising the integrity and function of myotubes. Besides, paclitaxel suppresses critical regulators of mitochondrial biogenesis, that is, the PGC‐1α/TFAM/NRF‐1 pathway [10], and suppresses crucial metabolic sensors such as SIRT1 and AMPK [11, 12]. Concurrently, it activates PDK4, which promotes metabolic rigidity and contributes to muscle atrophy [13, 14]. Overall, all these alterations compromise glucose and lipid metabolism, deplete glycogen stores, and hamper the metabolic plasticity of the muscle.

The mitochondrial dysfunction induced by paclitaxel generates tightly interwoven pathological outcomes such as hyperactive oxidative stress and cellular senescence induction while further resulting in elevated quantities of ROS, particularly mitochondrial superoxide, but lowering critical antioxidant defenses like superoxide dismutase (SOD) and glutathione (GSH) [8, 15, 16]. Such oxidative stress, largely a secondary consequence of mitochondrial injury, plays a role in muscle injury. Besides these effects, paclitaxel also disrupts anabolic signaling pathways involved in the maintenance and repair of muscle. Specifically, the inhibition of IGF1R signaling interrupts the PI3K/Akt axis, a central regulator of muscle growth, differentiation, and structural integrity [17, 18], leading to further muscle wasting.

Growing evidence suggests that cellular senescence is a central contributor to skeletal muscle degeneration due to chemotherapy. Senescent muscle cells, influenced by pathways such as mitochondrial impairment, chronic DNA damage, and persistent stress signaling, become irreversibly arrested in their growth, evidenced by cyclin‐dependent kinase inhibitors such as p21 upregulation, proinflammatory NFκB signaling activation, and secretion of a cocktail of inflammatory mediators termed the senescence‐associated secretory phenotype (SASP), which includes IL‐6 [19, 20]. These SASP factors cause inflammation locally, disrupt the niche of muscle stem cells, and impair regenerative ability [21, 22]. Mitochondrial pathology is both an initiator and amplifier of this senescent phenotype, and ROS‐induced damage hastens cellular aging further [23]. Among the key mediators, p21 has been a key effector to connect cellular stress with tissue‐level muscle breakdown [19].

**FIGURE 1:**
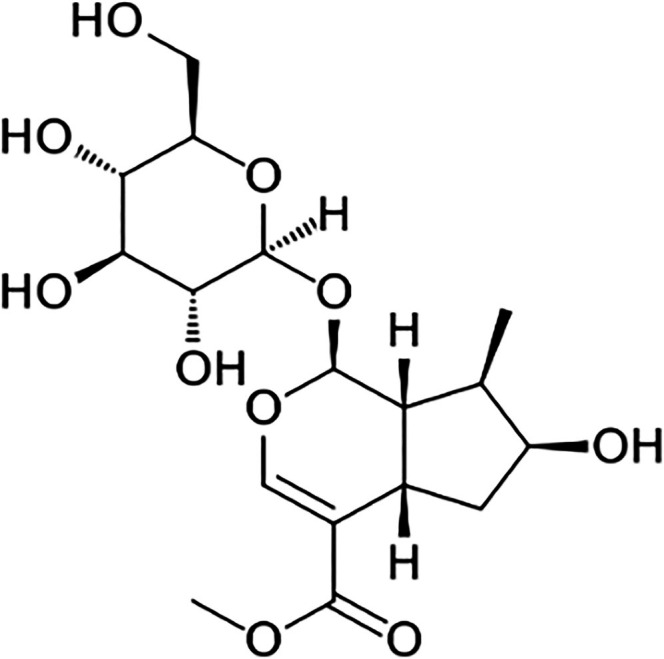
Chemical structure of loganin.

Given the multifactorial etiology of paclitaxel‐induced myopathy, treatment agents with the potential to simultaneously target mitochondrial damage, oxidative stress, and senescence are a priority. Natural bioactive compounds with multi‐targeted protective properties have gained attention as promising candidates where multitargeted natural bioactive molecules have been identified as hopeful leads. Loganin (Figure 1), a glycoside iridoid derived from *Cornus officinalis* , possesses antioxidant, anti‐inflammatory, and metabolic regulation activities in various disease models. It has also been discovered to augment mitochondrial function, boost redox balance, and modulate stress‐response pathways [24, 25, 26]; however, the protective capacity in chemotherapy‐induced skeletal muscle injury is unknown.

Herein, we investigated the therapeutic potential of loganin in mitigating paclitaxel‐induced skeletal muscle toxicity. Using C2C12 cells as an in vitro model system, we wanted to examine the effects of loganin on mitochondrial stability, oxidative stress, energy metabolism, and cellular senescence with the aim of demarcating the mechanistic basis for loganin's protective role and evaluating its potential as a therapeutic intervention against chemotherapy‐induced sarcopenia.

## 2 Methods

### 2.1 Materials

Loganin (≥ 97% purity by HPLC), DMSO, 2′,7′‐dichlorodihydrofluorescein diacetate (H_2_DCF‐DA), Senescence‐Associated β‐Galactosidase (SA‐β‐gal) Staining Kit, and MTT reagent were obtained from Sigma‐Aldrich (St. Louis, MO, USA). The LDH cytotoxicity assay kit was obtained from G‐Biosciences (St. Louis, MO, USA), Luminescent ATP and GSH Assay Kits from Abcam (Cambridge, MA, USA), and the SOD Activity Kit from Enzo Biochem (Farmingdale, NY, USA). RNeasy Mini and QIAamp DNA kits were obtained from Qiagen (Germantown, MD, USA), JC‐1, MitoSOX Red, Fast SYBR Green Master Mix, DMEM, culture supplements, Reverse Transcription Kit, T‐PER tissue protein extraction reagent, and NE‐PER nuclear and cytoplasmic extraction kit from Thermo Fisher Scientific (Waltham, MA, USA); and SDS‐PAGE reagents from Bio‐Rad (Hercules, CA, USA) with PVDF membranes/ECL reagents from Millipore (Billerica, MA, USA). Primary antibodies: β‐actin (catalog number: MAB1501), myosin heavy chain (MyHC) (catalog number: MAB4470) were acquired from R&D Systems (Minneapolis, MN, USA), PGC‐1α (Novus, Colorado, CO, USA, catalog number: NBP1‐04676), lamin B (catalog number: 12987‐1‐AP), TFAM (catalog number: 22586‐1‐AP), NRF‐1 (catalog number: 12482–1‐AP), and p21 (catalog number: 10355‐1‐AP) from Proteintech (Chicago, IL, USA). NF‐κB was supplied by iReal Biotechnology (Hsinchu, Taiwan, catalog number: IR137‐549); α‐tubulin by GeneTex (Hsinchu, Taiwan, catalog number: GTX112141); whereas IGF1R (catalog number: 3027), phospho‐AMPK (catalog number: 2535), AMPK (catalog number: 5832), SIRT1 (catalog number: 9475), and phospho‐NFκB (catalog number: 3033) were all acquired from Cell Signaling (Hsinchu, Taiwan). Secondary antibodies: anti‐rabbit IgG‐HRP (catalog number: GTX213110‐01), anti‐mouse IgG‐HRP (catalog number: GTX213111‐01) were obtained from GeneTex (Hsinchu, Taiwan), and Alexa Fluor 488‐conjugated goat anti‐mouse IgG (catalog number: A28175) from Thermo Fisher Scientific (Waltham, MA, USA).

### 2.2 Cell Culture and Drug Treatment

C2C12 myoblasts (CVCL_0188; Bioresource Collection and Research Center, Hsinchu, Taiwan) were cultured in DMEM with 10% FBS and antibiotics (100 U/mL penicillin, 100 μg/mL streptomycin) at 37°C, 5% CO_2_. C2C12 myoblasts were differentiated into myotubes to better replicate mature skeletal muscle physiology as myotubes more accurately model muscle fibers' functional and metabolic responses during chemotherapy‐induced toxicity. For differentiation, cells at ~80% confluence were cultured in DMEM with 2% horse serum for 6 days. To investigate the direct effects of loganin, C2C12 myotubes were treated with vehicle (0.1% DMSO), paclitaxel (0.01–1 μM) or loganin (0.01–1 μM) for 24 h. For cytoprotection assays, myotubes were pretreated with vehicle (0.1% DMSO) or loganin (0.01–1 μM) for 1 h before paclitaxel (50 nM) treatment for 24 h. To assess senescence, cells were treated with paclitaxel (50 nM) for 48 h with or without loganin pretreatment (0.01–1 μM).

### 2.3 MTT and LDH Assays

LDH release was assessed from culture supernatants using the LDH Assay Kit and read at 490 nm. MTT (0.5 mg/mL) was added to fresh medium for 2 h at 37°C. Formazan was dissolved in DMSO, and absorbance was measured at 540 nm.

### 2.4 RNA Isolation and qPCR

RNA was isolated (RNeasy Mini Kit), quantified, and reverse‐transcribed (High‐Capacity cDNA Kit). qPCR was performed using SYBR Green Master Mix, normalized to *Gapdh* and analyzed using the 2^−ΔΔC*q*^ method [27]. Primer sequences were: *Cdkn1a*: forward: 5′‐TTGTCGCTGTCTTGCACTCT‐3′, reverse: 5′‐TCTCTTGCAGAAGACCAATC‐3′, *Il6*: forward: 5′‐TGAACAACGATGATGCACTTG‐3′, reverse: 5′‐CTGAAGGACTCTGGCTTTGTC‐3′, *Gapdh*: forward: 5′‐CCCACTCTTCCACCTTCGAT‐3′, reverse: 5′‐CTTGCTCAGTGTCCTTGCTG‐3′.

### 2.5 mtDNA Content Assays

DNA was extracted using the QIAamp DNA mini kit according to the manufacturer's protocols. Mitochondrially encoded cytochrome c oxidase II (*Mt‐co2*) level was quantified by qPCR and normalized to 18S ribosomal RNA (18S rRNA). Primer sequences were: *Mt‐co2*: forward: 5′‐ATAACCGAGTCGTTCTGCCA‐3′, reverse: 5′‐GCTTGATTTAGTCGGCCTGG‐3′, 18S: forward: 5′‐CTCAACACGGGAAACCTCAC‐3′, reverse: 5′‐CGCTCCACCAACTAAGAACG‐3′.

### 2.6 Western Blotting

Proteins were extracted (T‐PER/NE‐PER), separated by SDS‐PAGE, transferred to PVDF membranes, probed with primary antibodies overnight at 4°C, then converted into HRP‐conjugated secondaries. Bands were visualized using ECL and quantified with ImageJ.

### 2.7 Measurement of Mitochondrial Membrane Potential (∆Ψm)

ΔΨm was measured using JC‐1 dye (2 μM, 30 min at 37°C). Red/green fluorescence ratio was read at 540/570 and 495/520 nm (excitation/emission), respectively.

### 2.8 ATP Quantification

Per the manufacturer's instructions, ATP levels were assessed using the Luminescent ATP Detection Kit, with luminescence measured via the BioTek microplate reader.

### 2.9 Measurement of ROS

Cells were incubated with H_2_DCF‐DA (10 μM, 30 min, 37°C), and fluorescence was read at 495/520 nm. DCF fluorescence, indicative of ROS levels, was measured using the BioTek microplate reader at 495/520 nm (excitation/emission). Cells were incubated with MitoSOX Red (5 μM, 15 min, 37°C). Mitochondrial superoxide levels were assessed by MitoSOX fluorescence using the BioTek microplate reader at 510/580 nm (excitation/emission).

### 2.10 Determination of GSH Content

According to the manufacturer's instructions, GSH levels were quantified with a Colorimetric Assay Kit. Lysates were deproteinized and incubated with the reaction mix for 30 minutes. Fluorescence was read at 340/420 nm and compared to GSH standards.

### 2.11 Determination of SOD Activity

SOD activity was measured from lysates using a commercial SOD Activity Assay Kit according to the manufacturer's instructions. Absorbance at 450 nm was used to calculate activity as percent inhibition, relative to a standard curve.

### 2.12 Periodic Acid–Schiff (PAS) Staining

Myotubes were fixed, oxidized with 1% periodic acid, and stained with Schiff's reagent for 20 min. Glycogen accumulation was imaged and quantified via ImageJ.

### 2.13 Immunofluorescence Staining

Fixed myotubes were permeabilized and blocked, stained for MyHC (1:1000) with Alexa Fluor 488 secondary antibody, and counterstained with DAPI. Myotube number and length were quantified in five random fields (≥ 60 myotubes/group) using ImageJ.

### 2.14 SA‐β‐Gal Staining

Senescence was assessed using the SA‐β‐gal staining kit according to the manufacturer's protocol. Stained (blue) and unstained cells were imaged at ×10 magnification and quantified.

### 2.15 Statistical Analysis

All experiments were conducted by operators blinded to the treatment groups. Data are presented as mean ± SEM. Statistical analysis was performed using GraphPad Prism 10. Statistical tests used are specified in the figure captions. Significance was accepted at *p* < 0.05. Data were normalized to percentage matched controls to reduce unwanted sources of variation, except for myotube number.

## 3 Results

### 3.1 Loganin Protects C2C12 Myotubes From Paclitaxel‐Induced Cytotoxicity and Enhances Mitochondrial Biogenesis Signaling

**FIGURE 2:**
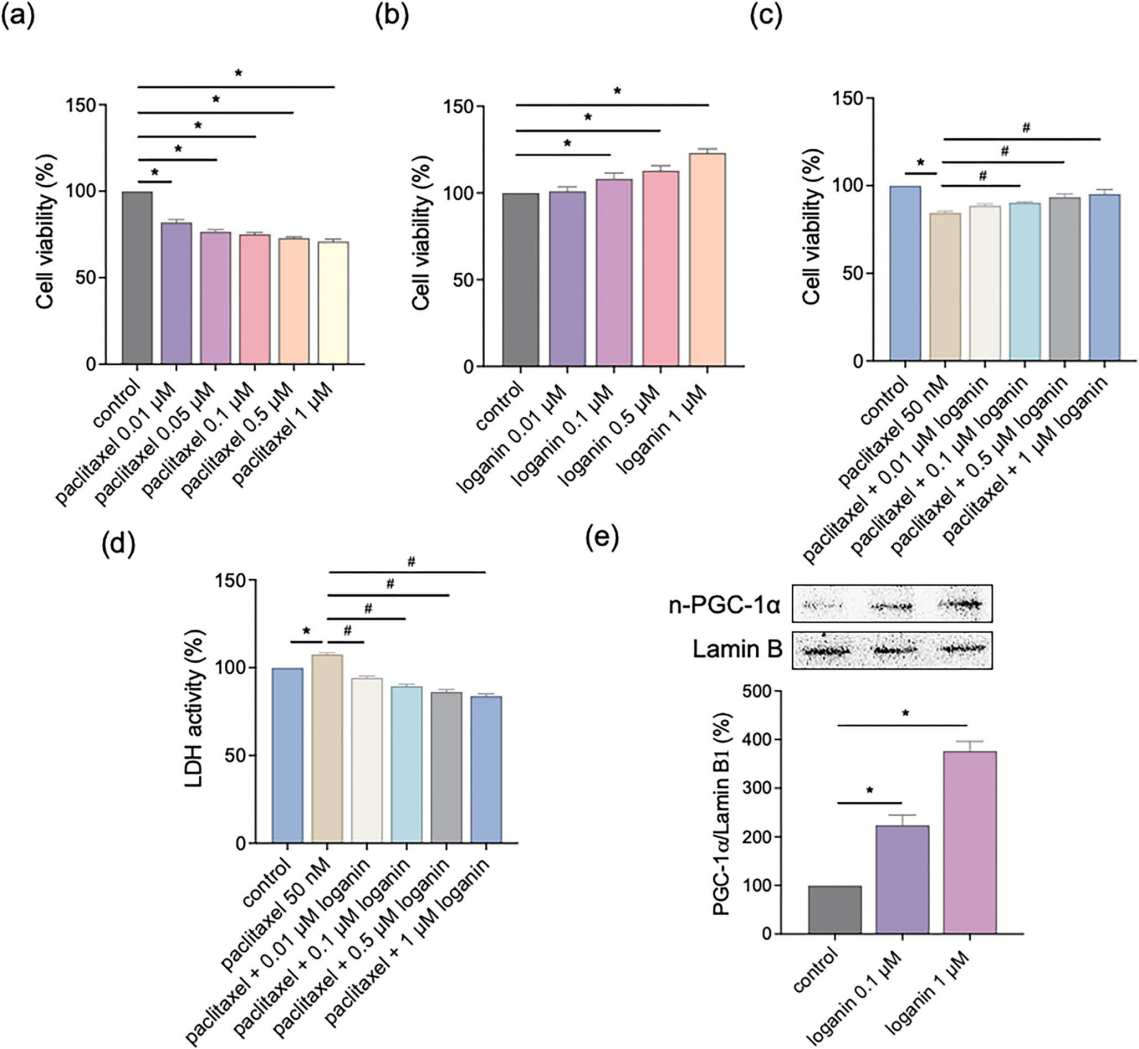
Loganin protects C2C12 myotubes from paclitaxel‐induced cytotoxicity and enhances nuclear PGC‐1α levels. Cell viability assessed by MTT assay after treatment with paclitaxel (0.01–1 μM, as indicated) for 24 h (a) or loganin (0.01–1 μM, as indicated) (b). (c) Cell viability was assessed by MTT assay after treatment with paclitaxel (50 nM) in the presence or absence of loganin (0.01–1 μM, as indicated) for 24 h. (d) LDH release after treatment with paclitaxel (50 nM) in the presence or absence of loganin (0.01–1 μM, as indicated) for 24 h. (e) Cells were treated with loganin (0.1 and 1 μM) for 24 h. Protein expression of nuclear PGC‐1α was measured by Western blotting analysis. Densitometry analyses are presented relative to a loading control and expressed as percentages of the matched control groups. Error bars represent the mean ± SEM (*n* = 3). *p < 0.05 versus control, ^#^ *p* < 0.05 versus paclitaxel alone (one‐way ANOVA with Tukey's multiple comparisons test).

To quantify the cytotoxic action of paclitaxel and identify the protective role of loganin, we first performed an MTT assay. Paclitaxel (0.01–1 μM) exhibited concentration‐dependent inhibition of cell viability significantly, as demonstrated by its cytotoxicity against differentiated C2C12 myotubes (Figure 2a). In contrast, loganin‐alone (0.01–1 μM) treatment did not have any adverse effect on cell viability, indicating its safety under these concentrations (Figure 2b). Notably, co‐treatment of loganin (0.1 and 1 μM) significantly increased cell viability in paclitaxel‐treated cells (Figure 2c). This cytoprotective effect was further substantiated by LDH release assays, wherein the paclitaxel‐triggered increase in extracellular LDH, a marker for cell membrane damage, was significantly inhibited by loganin co‐treatment (Figure 2d). To begin elucidating the mechanisms underlying such protection, we examined a key component of mitochondrial biogenesis signaling. Nuclear PGC‐1α levels, a master transcriptional coactivator of mitochondrial biogenesis, were significantly induced by loganin (0.1 and 1 μM) after 24‐h treatment (Figure 2e). The result suggests that loganin's action on the mitochondrial biogenesis pathway contributes to its reversal of paclitaxel‐induced cytotoxicity.

### 3.2 Loganin Restores Mitochondrial Function in Paclitaxel‐Treated Myotubes

**FIGURE 3:**
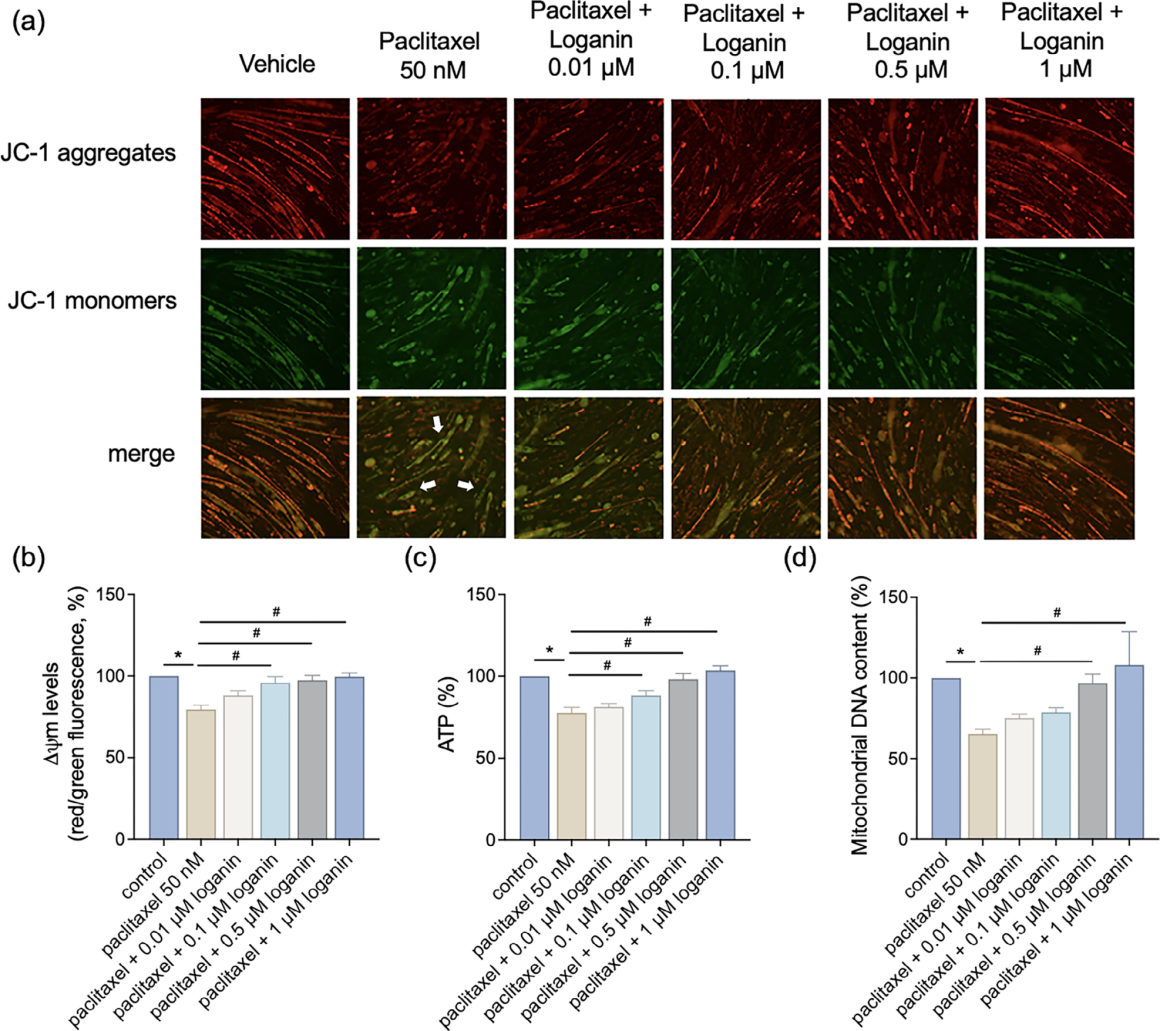
Loganin restores mitochondrial function in paclitaxel‐treated myotubes. Cells were treated with paclitaxel (50 nM) in the presence or absence of loganin (0.01–1 μM, as indicated) for 24 h. (a) Representative immunofluorescence images of mitochondrial membrane potential (ΔΨm) using JC‐1 dye. Red fluorescence indicates polarized mitochondria, while green fluorescence indicates depolarized mitochondria. White arrows highlight cells exhibiting a loss of mitochondrial membrane potential (decreased red/green ratio) in the paclitaxel group. Scale bar = 100 μm. (b) Quantification of JC‐1 fluorescence intensity. (c) Cellular ATP production levels. (d) mtDNA content. Error bars represent the mean ± SEM (*n* = 3). *p < 0.05 versus control, ^#^ *p* < 0.05 versus paclitaxel alone (one‐way ANOVA with Tukey's multiple comparisons test).

To determine whether loganin preserves mitochondrial integrity in paclitaxel‐treated myotubes, we measured mitochondrial membrane potential (ΔΨm), ATP production, and mtDNA levels. Immunofluorescence staining revealed that 0.05 μM paclitaxel resulted in extensive loss of ΔΨm, indicated by reduced fluorescence intensity (Figure 3a,b), which was reversed by loganin in a concentration‐dependent manner, with maximal improvement at 1 μM loganin (Figure 3b). ATP measurement revealed that paclitaxel substantially suppressed cellular energy generation. However, loganin concentration‐dependently increased ATP levels in paclitaxel‐treated cells (Figure 3c). Besides, mtDNA content, as an indicator of mitochondrial quantity and integrity, was severely decreased by paclitaxel. Co‐treatment with 0.5 and 1 μM loganin restored mtDNA content (Figure 3d), which implies that loganin preserves mitochondrial bioenergetic function under stress induced by chemotherapy.

### 3.3 Loganin Attenuates Paclitaxel‐Induced Oxidative Stress

**FIGURE 4:**
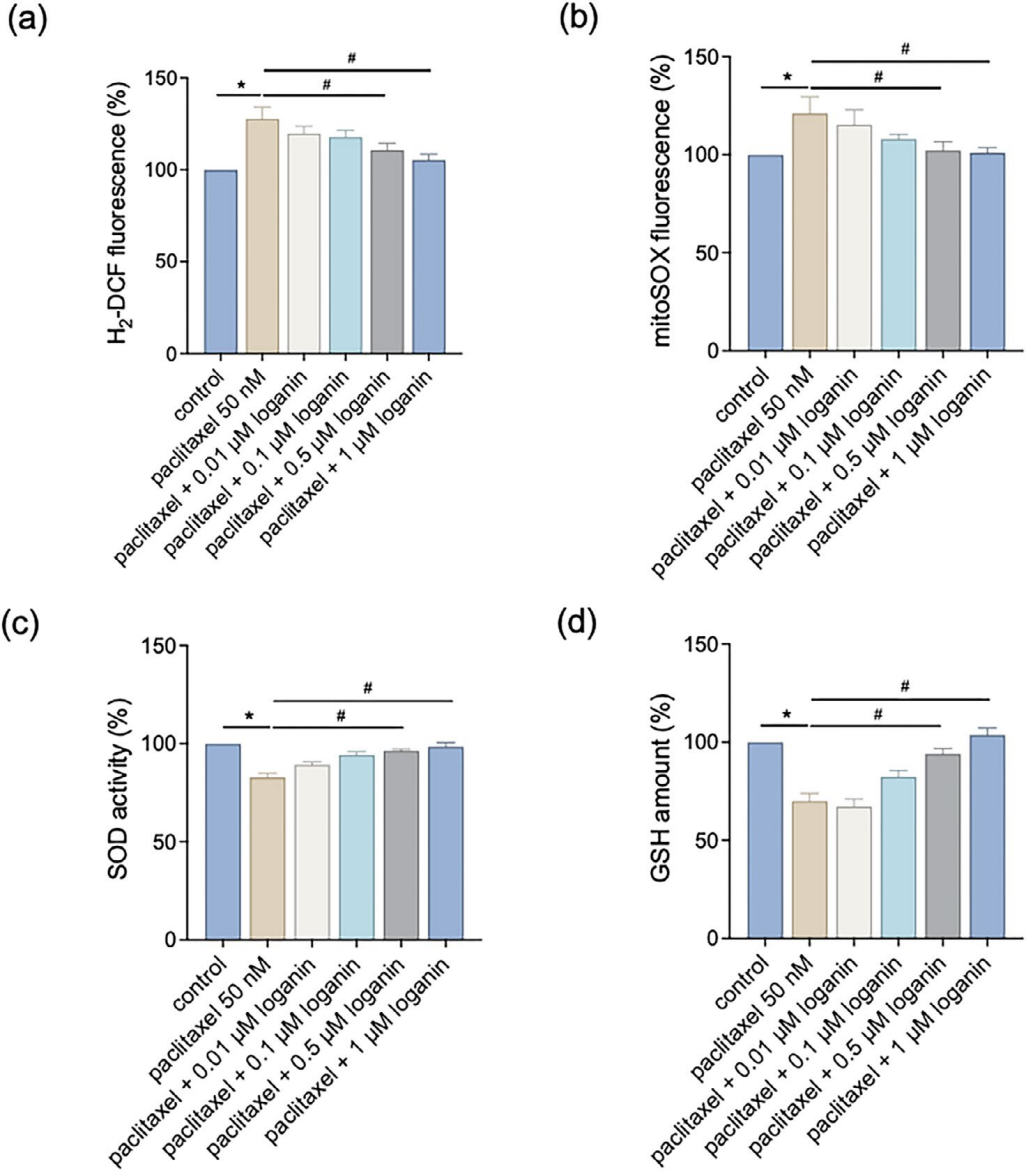
Loganin attenuates paclitaxel‐induced oxidative stress and enhances antioxidant capacity. C2C12 myotubes were treated with paclitaxel (50 nM) in the presence or absence of loganin (0.01–1 μM, as indicated) for 24 h. (a) Intracellular ROS levels by DCFDA fluorescence. (b) Mitochondrial ROS (superoxide) levels by MitoSOX fluorescence. (c) SOD activity. (d) Intracellular GSH content. Error bars represent the mean ± SEM (*n* = 3). *p < 0.05 versus control, ^#^ *p* < 0.05 versus paclitaxel alone (one‐way ANOVA with Tukey's multiple comparisons test).

In order to investigate the role of oxidative stress in paclitaxel‐induced mitochondrial impairment and its inhibition by loganin, we analyzed ROS accumulation and antioxidant defense mechanisms. DCFDA staining showed increased total ROS after paclitaxel treatment, which was significantly downregulated by loganin in a concentration‐dependent manner (Figure 4a). MitoSOX staining for mitochondrion‐specific ROS also showed increased superoxide production after paclitaxel treatment; this increase was markedly reduced by loganin co‐treatment (Figure 4b). Paclitaxel also reduced the expression of the antioxidant enzyme SOD and GSH content. Loganin treatment could reverse these effects, with high restoration of SOD protein levels and GSH content (Figure 4c,d), suggesting restoration of the cellular antioxidant defense system.

### 3.4 Loganin Enhances Key Regulators of Mitochondrial Biogenesis and Metabolism

**FIGURE 5:**
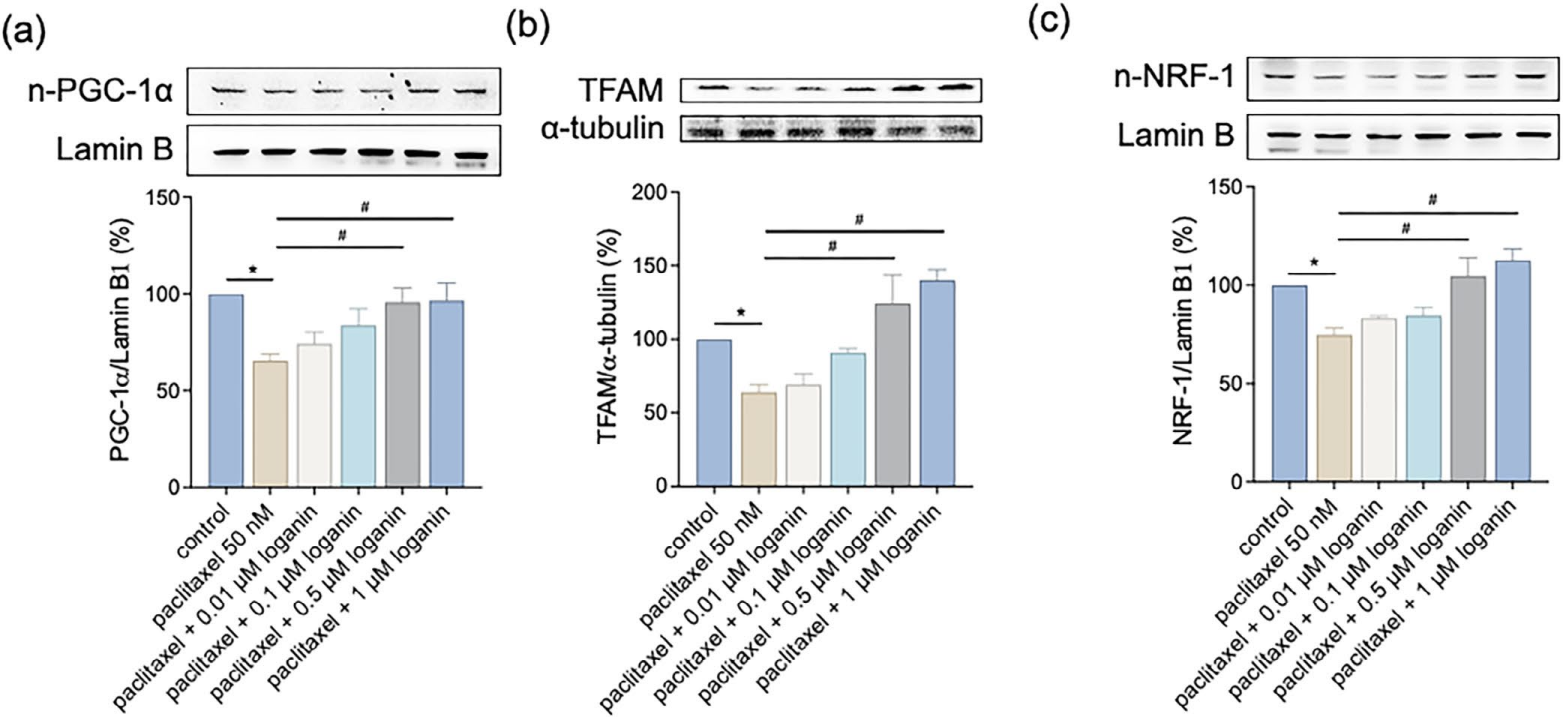
Loganin restores the expression of mitochondrial biogenesis regulators in paclitaxel‐treated myotubes. Cells were treated with paclitaxel (50 nM) in the presence or absence of loganin (0.01–1 μM, as indicated) for 24 h. Protein expressions of (a) PGC‐1α, (b) TFAM, and (c) NRF‐1 were measured by western blotting analysis. Densitometry analyses are presented relative to a loading control and expressed as percentages of the matched control groups. Error bars represent the mean ± SEM (*n* = 3). *p < 0.05 versus control, ^#^ *p* < 0.05 versus paclitaxel alone (one‐way ANOVA with Tukey's multiple comparisons test).

**FIGURE 6:**
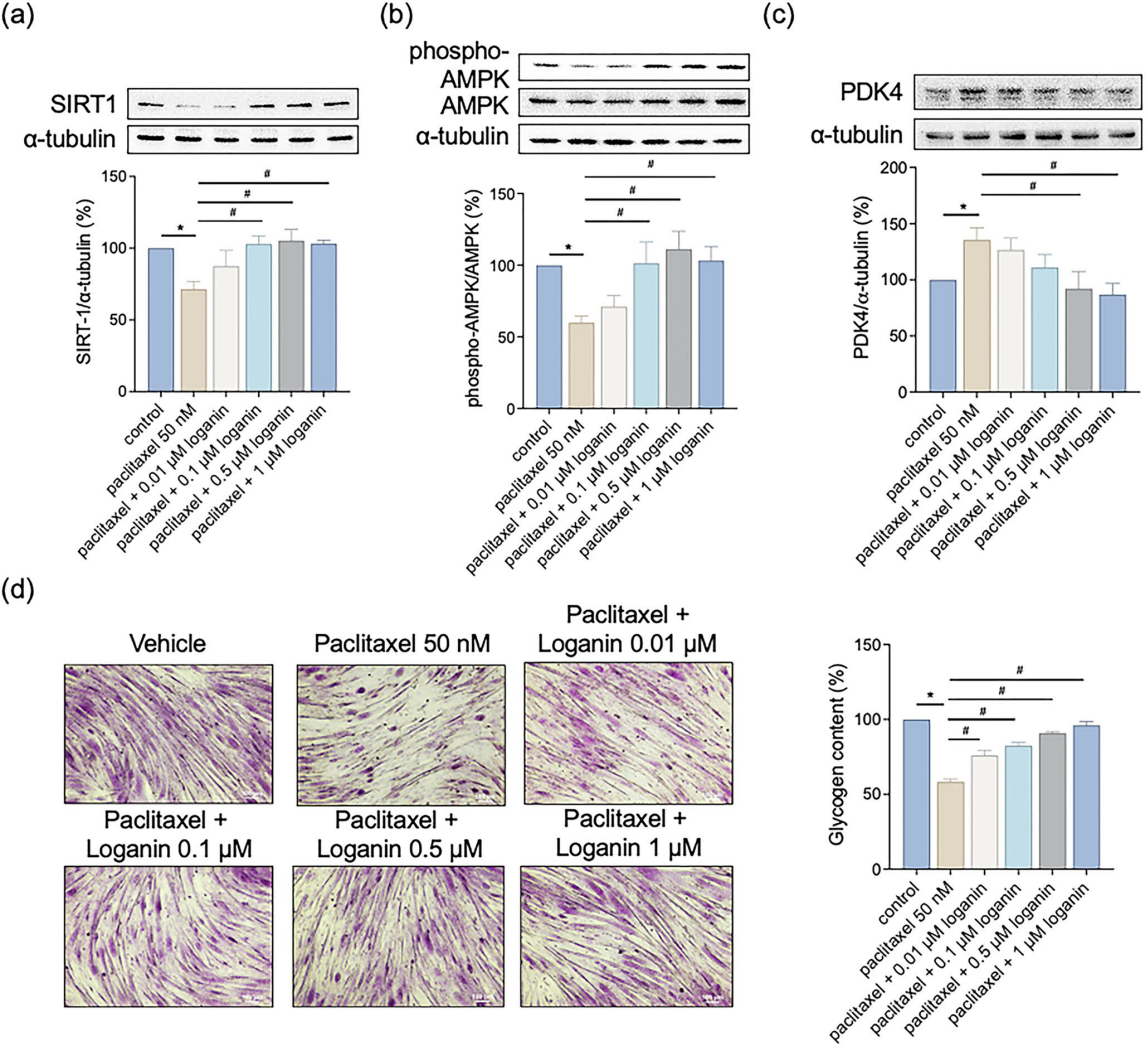
Loganin reverses paclitaxel‐induced dysregulation of metabolic regulators and restores glycogen content. C2C12 myotubes were treated with paclitaxel (50 nM) in the presence or absence of loganin (0.01–1 μM, as indicated) for 24 h. Protein expressions of (a) SIRT1, (b) AMPK, and (c) PDK4 were measured by western blotting analysis. (d) Cellular glycogen content. For Western blots (a–c), densitometry analyses are presented relative to a loading control (a) or total AMPK (b) and expressed as percentages of the matched control groups. Error bars represent the mean ± SEM (*n* = 3). *p < 0.05 versus control, ^#^ *p* < 0.05 versus paclitaxel alone (one‐way ANOVA with Tukey's multiple comparisons test).

To further elucidate loganin's involvement in mitochondrial regulation, we assessed the expression of critical proteins that regulate mitochondrial rejuvenation. Paclitaxel significantly suppressed expression levels of PGC‐1α, TFAM, and NRF‐1, while loganin co‐treatment restored these expression levels (Figure 5a–c), indicating reactivation of mitochondrial renewal pathways. We also analyzed critical metabolic modulators and found paclitaxel repressed SIRT1 and AMPK expression while causing PDK4 upregulation, pointing towards impaired metabolic homeostasis. Loganin significantly augmented SIRT1 and AMPK expression and corrected PDK4 levels in paclitaxel‐treated cells (Figure 6a–c), suggesting improved energy regulation. Glycogen content, depleted by paclitaxel, was significantly restored by loganin co‐treatment (Figure 6d), further supporting metabolic recovery.

### 3.5 Loganin Restores IGF1R Signaling and Preserves Myotube Integrity

**FIGURE 7:**
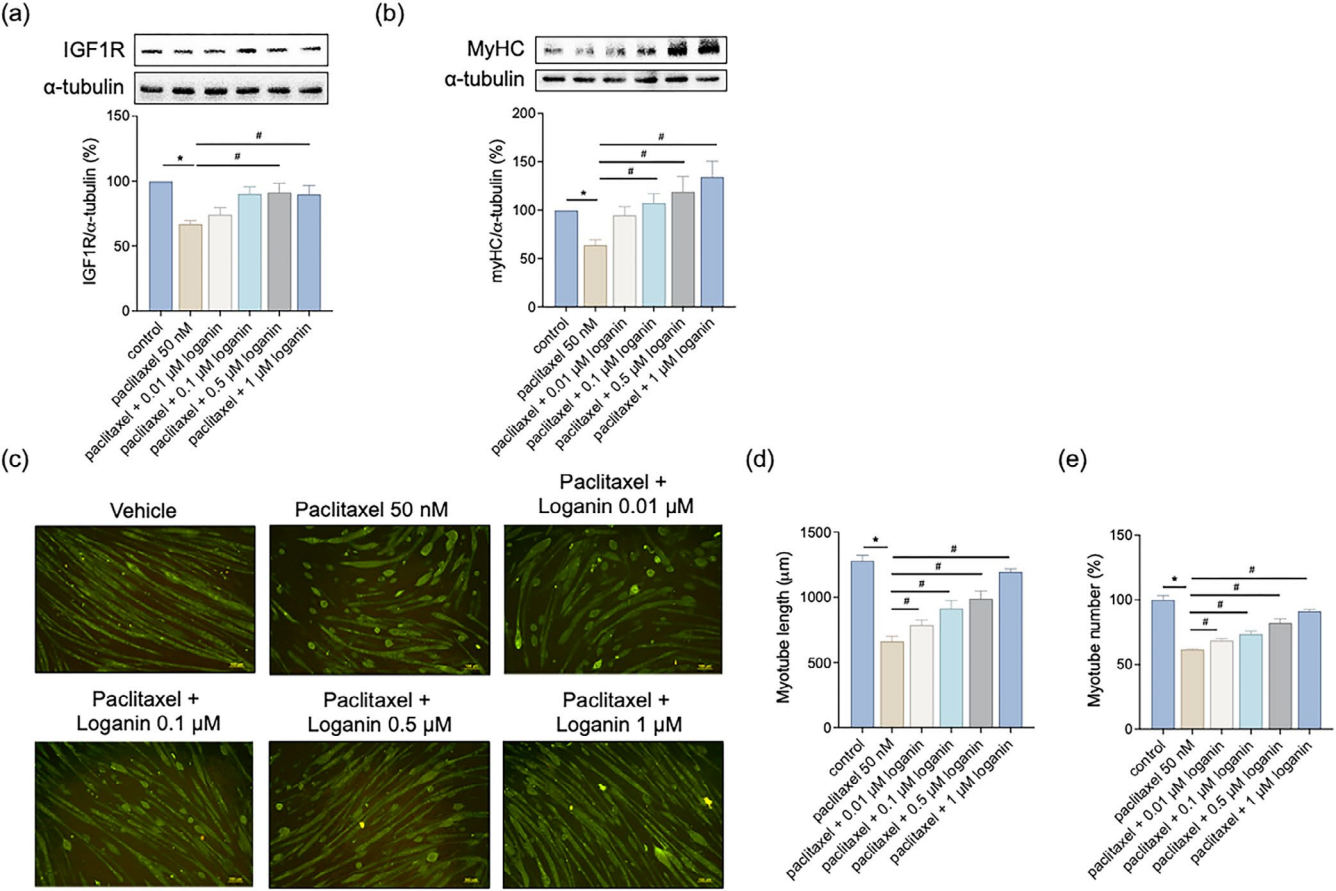
Loganin preserves myotube integrity and IGF1R signaling against paclitaxel‐induced damage. Cells were treated with paclitaxel (50 nM) in the presence or absence of loganin (0.01–1 μM, as indicated) for 24 h. Protein expressions of (a) IGF1R and (b) MyHC were measured by Western blotting analysis. (c) Representative immunofluorescence images of myotube morphology and MyHC staining. Scale bar = 100 μm. (d) Quantification of myotube length. (e) Quantification of myotube number. For Western blots (a, b), densitometry analyses are presented relative to a loading control and expressed as percentages of the matched control groups. Error bars represent the mean ± SEM (*n* = 3). *p < 0.05 versus control, ^#^ *p* < 0.05 versus paclitaxel alone (one‐way ANOVA with Tukey's multiple comparisons test).

To assess the impact of loganin on muscle structural integrity, we contrasted IGF1R expression and morphological features of myotubes. Paclitaxel significantly suppressed IGF1R expression, whereas co‐treatment with loganin restored its levels (Figure 7a), reflecting the rescue of anabolic signaling. Furthermore, Western blot and immunofluorescence data demonstrated that paclitaxel suppressed the expression of MyHC and disrupted myotube structure. These cytotoxic effects were alleviated by loganin, which preserved MyHC expression (Figure 7b,c) and significantly increased myotube length and number (Figure 7d,e). These findings indicate that loganin preserves muscle architecture in response to chemotherapeutic damage.

### 3.6 Loganin Suppresses Paclitaxel‐Induced Cellular Senescence and Associated Inflammation

**FIGURE 8:**
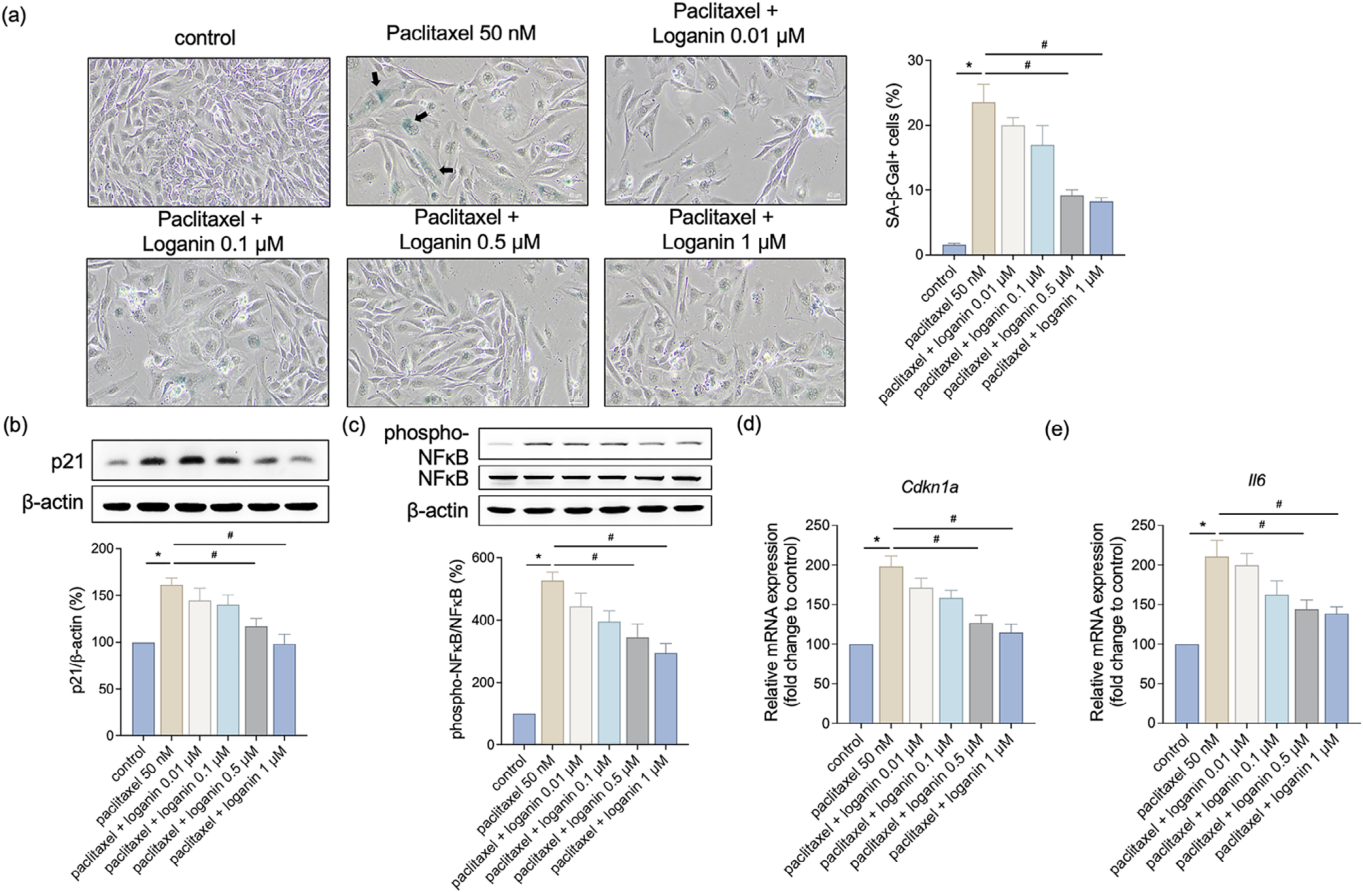
Loganin suppresses paclitaxel‐induced cellular senescence and associated inflammation. C2C12 cells were treated with paclitaxel (50 nM) in the presence or absence of loganin (0.01–1 μM, as indicated) for 24 h. (a) Representative images of SA‐β‐galactosidase (SA‐β‐gal) staining. Blue‐stained cells indicate senescent cells. Black arrows denote SA‐β‐gal‐positive cells with characteristic enlarged and flattened morphology in the paclitaxel group. Quantification of SA‐β‐gal‐positive cells is shown on the right. Scale bar = 40 μm. Protein expressions of (b) p21 and (c) phospho‐NFκB were measured by western blotting analysis. (d) Relative mRNA levels of *Cdkn1a* assessed by qPCR. (e) Relative mRNA levels of *Il6* assessed by qPCR. For Western blots (b, c), densitometry analyses are presented relative to a loading control (b) or total NFκB (c) and expressed as percentages of the matched control groups. For qPCR (d, e), data are normalized to Gapdh and expressed as percentages of the matched control groups. Error bars represent the mean ± SEM (*n* = 3). *p < 0.05 versus control, ^#^ *p* < 0.05 versus paclitaxel alone (one‐way ANOVA with Tukey's multiple comparisons test).

To know whether loganin impacts paclitaxel‐induced cellular senescence, we examined SA‐β‐gal staining and key markers of the SASP. Treatment with paclitaxel significantly increased the percentage of SA‐β‐gal‐positive cells. These senescent cells exhibited characteristic blue staining, indicated by arrows in Figure 8a. Pretreatment with loganin significantly lowered this ratio (Figure 8a). Western blot and gene expression analyses also demonstrated that paclitaxel induced p21, phospho‐NFκB, *Cdkn1a*, and *Il6*, known markers of senescence and inflammation. Co‐treatment with loganin lowered these markers significantly (Figure 8b–e), suggesting its suppressive activities on senescence and SASP. The restoration of IGF1R signaling and reduction in oxidative stress noted might partially mediate the anti‐senescent and anti‐inflammatory effects of loganin.

## 4 Discussion

The current research provides compelling evidence that loganin possesses multifunctional protective effects against paclitaxel‐induced skeletal muscle toxicity. Through the protection of mitochondrial function, regulation of metabolic regulators, restoration of IGF1R signaling, alleviation of oxidative stress, and suppression of cellular senescence, loganin might be a potential candidate agent for chemotherapy‐induced muscle atrophy. The latter condition is a serious clinical concern, particularly in the extensive use of paclitaxel in cancer therapy.

Mitochondria play a central role in cellular bioenergetics, redox homeostasis, and the maintenance of muscle. Mitochondrial defects caused by paclitaxel, such as reduced content and function, lead to metabolic failure and oxidative damage in muscle cells. Indeed, chemotherapeutic drugs such as paclitaxel can induce mitochondrial loss and production of higher levels of oxidants in skeletal muscle, leading to atrophy and dysfunction [9, 15]. Our findings demonstrate that loganin treatment suppressed paclitaxel‐caused ROS accumulation, preserved mitochondrial membrane potential, and restored ATP synthesis, indicative of enhanced mitochondrial integrity and resistance. These were accompanied by upregulation of the mitochondrial biogenesis and antioxidant defense master regulators PGC‐1α, TFAM, and NRF1, allowing for mitochondrial DNA replication and transcription. Paclitaxel significantly downregulated PGC‐1α expression (Figure 5a), consistent with findings that chemotherapy suppresses mitochondrial biogenesis regulators. This might reflect impaired upstream signaling through AMPK or SIRT1, both known to activate PGC‐1α transcriptionally. The reduction in PGC‐1α likely contributes to paclitaxel‐induced mitochondrial dysfunction. Our findings indicate that loganin supports mitochondrial renewal and functional restoration in muscle cells. These same enhancements to mitochondrial biogenesis have been observed with other natural compounds; for instance, daidzein regulates this process in muscle cells via a SIRT1‐related mechanism [28]. Successful augmentation of mitochondrial biogenesis and quenching ROS is at the heart of skeletal muscle homeostasis under chemotherapeutic insult. To further determine the origin of ROS, both DCFDA (for general ROS) and MitoSOX (for mitochondrial superoxide) staining were employed. The markedly increased MitoSOX signaling in paclitaxel‐treated cells suggests that mitochondria are a primary source of ROS generation; however, the parallel elevation of DCFDA signal indicates that both mitochondrial and cytosolic compartments might contribute to the oxidative burden. Additional studies using mitochondrial‐targeted ROS scavengers will be necessary to delineate the contribution of different ROS sources more precisely.

This is supported by experiments showing that enhancing mitochondrial quality control and antioxidant defenses can be preventive against muscle wasting and dysfunction in various disease and stress models [29, 30, 31]. Notably, loganin treatment at 0.1–1 μM concentrations had augmented cell viability in the MTT assay (Figure 2b), which could indicate a proliferative effect. Although the MTT assay specifically measures mitochondrial metabolic activity, increased viability could also reflect higher proliferation. Nevertheless, given that our experiments were carried out in post‐mitotic differentiated C2C12 myotubes, which do not readily proliferate, the probability of an increase in cell number playing a role in the responses observed is low. Instead, the increase in MTT signaling is better reasoned due to heightened mitochondrial activity and bioenergetic function. Nevertheless, we cannot completely exclude the possibility that metabolic enhancement or a shift in cellular activity contributed to the results.

SIRT1 and AMPK are critical energy sensors that stimulate mitochondrial function, increase oxidative metabolism, and maintain muscle integrity [11]. Our results indicate that treatment with loganin activated AMPK and SIRT1 signaling pathways, which are crucial for regulating energy homeostasis and mitochondrial function and improving metabolic efficiency. Activation of these pathways improves cellular energy balance and maintains muscle cell survival under stress. For comparison, the natural phytochemical ursolic acid activates mitochondrial biogenesis through the activation of AMPK and PGC‐1α in C2C12 myotubes, suggesting a potentially equivalent mechanism for loganin [32]. Moreover, loganin modulation of PDK4 expression might improve glucose oxidation, reduce lactic acid accumulation, and improve energy homeostasis in paclitaxel‐stressed myotubes. Enhanced glycogen storage in loganin‐treated cells is in line with this metabolic shift, and it represents improved substrate availability for energy production.

The IGF1R signaling pathway is essential in regulating muscle growth, differentiation, and maintenance of muscle mass [18, 33]. Disruption of this anabolic process is classically observed in muscle atrophy after various stress paradigms [34]. Although immediate evidence of paclitaxel inducing inhibition of IGF1R in skeletal muscle is limited, paclitaxel is known to undermine muscle integrity through mechanisms that may overlap with growth factor signaling [8, 35]. Our findings indicate that loganin reverses the loss of IGF1R expression, potentially stands behind the described increase in MyHC content, and improves muscle cell morphology and function. This is consistent with evidence demonstrating activation of the Akt/FOXO1 pathway, downstream of IGF1R, to induce proliferation of C2C12 myoblasts, highlighting the importance of IGF1R signaling to muscle cell function [36]. The intact number and length of myotubes of the loganin‐treated cultures also suggest its role in preserving the cytoskeletal integrity, most likely through increased anabolic signaling and ROS‐mediated structural protection.

Paclitaxel is known to induce premature cellular senescence, a state of permanent growth arrest characterized by elevated p21 (CDKN1A) expression and increased SA‐β‐gal activity [37]. This senescent phenotype contributes to a pro‐inflammatory microenvironment, SASP, marked by the secretion of cytokines such as IL‐6, IL‐17, and TNF‐α [38], that together jeopardize muscle regenerative capacity and augment tissue degeneration. In this regard, our findings demonstrate that loganin significantly reduces paclitaxel‐induced senescence in C2C12 cells, as evidenced by reduced p21 expression and reduced SA‐β‐gal staining. Furthermore, loganin suppressed NFκB phosphorylation and SASP‐related cytokine expression, including IL‐6, indicating its potential for suppressing chronic inflammation. Paclitaxel‐induced senescence was also accompanied by increased IL‐6 expression and NFκB activation, key components of the SASP. NFκB is a primary regulator of inflammatory cytokine production in cellular senescence, and its activation can worsen tissue damage and inflammation. Loganin's suppression of IL‐6 and NFκB suggests that it attenuates SASP, contributing to its anti‐inflammatory and anti‐senescent effects. Since oxidative stress has been identified as a true upstream inducer of senescence and NFκB activation [39, 40], the antioxidative activity of loganin is most likely an integral part of its anti‐senescent and anti‐inflammatory effect. These results show that loganin could prevent muscle aging and inflammation caused by chemotherapy, playing a role in maintaining muscle tissue homeostasis.

Current research indicates that loganin has significant protective effects against skeletal muscle toxicity induced by paclitaxel in C2C12 cells. It achieves this by restoring mitochondrial bioenergetics, reducing oxidative stress, modulating metabolic signaling pathways, preserving anabolic signaling, and inhibiting cellular senescence and related inflammation. These actions target multiple pathological features associated with chemotherapy‐induced muscle injury. Consequently, loganin may serve as a preventive supplement to chemotherapy, aimed at preserving muscle health. Further in vivo experiments are needed to confirm its efficacy and explore its potential clinical applications for preventing chemotherapy‐induced sarcopenia.

## Conflicts of Interest

The authors declare no conflicts of interest.

## Data Availability

The data that support the findings of this study are available from the corresponding author upon reasonable request.
